# Recurrent Multiple Dyselectrolytemias Secondary to Episodic Water Intoxication in a Young Lady: A Case Report

**DOI:** 10.7759/cureus.10665

**Published:** 2020-09-26

**Authors:** Georgia K Galloway, Sally Babiker, Samson O Oyibo

**Affiliations:** 1 General Medicine, Peterborough City Hospital, Peterborough, GBR; 2 Diabetes and Endocrinology, Peterborough City Hospital, Peterborough, GBR

**Keywords:** water intoxication, dyselectrolytemias, hyponatremia, euvolemia, polydipsia, sodium, potassium, multiple

## Abstract

Water intoxication is a life-threatening disorder accompanied by brain function impairment due to severe dilutional hyponatremia. We present a young woman who had multiple emergency admissions with severe dyselectrolytemias involving several electrolytes. Further assessment revealed a long history of chronic polydipsia and episodic water intoxication. Her serum electrolytes were normal after an overnight fluid fast. She had no further admissions after discussion and counseling concerning excessive water drinking. This case emphasizes the importance of obtaining an accurate fluid intake history in cases of hyponatremia and multiple electrolyte disturbances.

## Introduction

In cases of water intoxication, there can be a significant imbalance in serum electrolytes [1]. Furthermore, this disturbance results in hypo-osmolality, and the kidneys fail to excrete the excess free water. The end result can potentially cause a fatal cerebral disturbance [1].

For patients who present with multiple dyselectrolytemias, there are several different diagnoses that need to be considered. These include acute water intoxication, psychogenic polydipsia (accompanying anorexia nervosa, schizophrenia, personality disorders), beer potomania, Addisonian crisis, diabetic ketoacidosis, Conn’s syndrome, iatrogenic fluid excess during total parenteral nutrition, after ingestion of ecstasy (methylenedioxymethamphetamine), syndrome of inappropriate antidiuretic hormone secretion (SIADH), and diuretics abuse [2-4].

Here, we present a case of recurrent multiple electrolyte disturbances associated with significant water intoxication in a 26-year-old female. This clinical scenario highlights the importance of taking a thorough history before considering the underlying causes of recurrent electrolyte disturbance.

## Case presentation

Medical history and demographics

A 26-year-old female presented to the emergency department (ED) with a one-day history of feeling unwell, nausea, and vomiting. She had previous ED visits where she was found to have multiple electrolyte disturbances as well as symptoms of intermittent abdominal pain and vomiting. Her past medical history also included a diagnosis of abdominal migraines after extensive investigation as well as a past history of eating disorder. Her regular medications included propranolol 80 mg daily, omeprazole 40 mg daily, prochlorperazine 3 mg twice a day, and haloperidol 0.5 mg three times a day for intermittent nausea. The general examination was unremarkable. At the time of her visit, she weighed 53.0 kg (body mass index (BMI) 21.5 kg/m^2^).

Investigations

**Figure 1:**
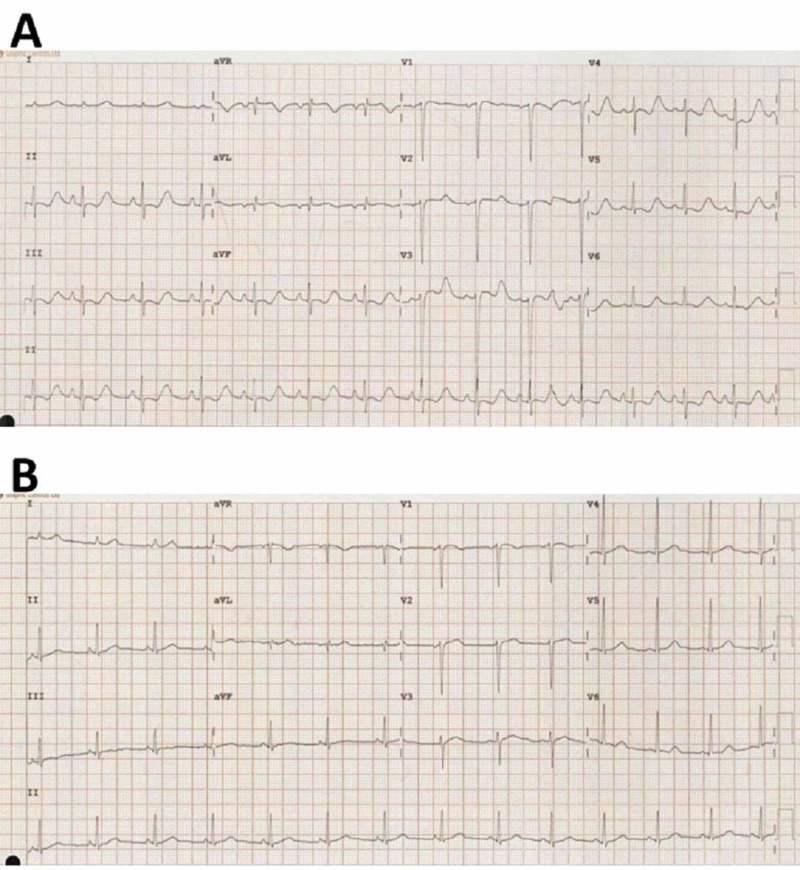
Serial electrocardiograms performed during patient's admission with dyselectrolytemias The initial electrocardiogram (A) showing inverted T-waves, Q-T interval prolongation, prominent U-waves, and mild ST depression caused by severe hypokalemia, and a follow-up electrocardiogram (B) showing complete resolution after correction of the hypokalemia.

Initial blood investigations revealed very low levels of multiple serum electrolytes with life-threatening hypokalemia. Urine investigations also revealed a low urine sodium level along with reduced urine osmolality (Table 1). An electrocardiogram demonstrated life-threatening features in keeping with severe hypokalemia (Figure 1).

**Table 1:** Initial blood and urine investigations at presentation Results demonstrate a global reduction in multiple serum and urine electrolytes and osmolality

Test	Normal value	Patient’s results
Serum sodium (mmol/L)	132-145	123
Serum potassium (mmol/L)	3.4-5.1	2.1
Serum creatinine (mmol/L)	45-84	37
Serum urea (mmol/L)	2.5-7.8	1.4
Serum chloride (mmol/L)	97-110	67
Serum corrected calcium (mmol/L)	2.20-2.60	2.65
Serum phosphate (mmol/L)	0.8-1.5	0.29
Serum magnesium (mmol/L)	0.7-1.0	0.6
Blood glucose (mmol/L)	4.0-7.0	5.4
Serum bicarbonate (mmol/L)	23-30	28
Serum thyroid stimulating hormone (mU/L)	0.3-4.2	0.66
Serum 9-am cortisol (nmol/L)	250-600	296
Serum cortisol after an adrenocorticotrophic stimulation test (nmol/L)	>450	601
Serum osmolality (mOsm/kg)	280-300	237
Hemoglobin (g/L)	115-165	147
Hematocrit (L/L)	0.36-0.46	0.390
Urine osmolality (mOsm/kg)	300-1100	47
Urine sodium (mmol/L)	40-220	17

Treatment

The patient was treated with intravenous potassium, phosphate, and magnesium in 0.9% sodium chloride solution while maintaining a 2 liter/day fluid restriction. Serum electrolytes returned to normal within 72 hours. She was discharged shortly afterwards with a short course of oral potassium.

Outcome and follow-up

The reason for her presentations remained undetermined because she denied excess fluid intake or a concurrent eating disorder. Historical results indicated chronic mild hypokalemia (range: 2.9-3.2 mmol/L) and hypochloremia (range: 67-94 mmol/L). Renin-aldosterone assay during normal potassium levels ruled out Conn’s syndrome. The patient’s serum electrolytes began to fall again but returned to normal after an overnight (12-hour) fluid fast. This confirmed reversibility of the patient's dyselectrolytemias (Table 2).

**Table 2:** Results of the overnight (12-hour) fluid fast Demonstrating a reversal of the patient’s multiple dyselectrolytemias after the overnight (12-hour) fluid fast. All results are chronically low before the overnight fluid fast and revert to normal thereafter.

Test	Before the fluid fast results	After the fluid fast results
Serum sodium (mmol/L)	132	143
Serum potassium (mmol/L)	3.4	4.8
Serum creatinine (mmol/L)	47	62
Serum chloride (mmol/L)	94	102
Serum osmolality (mOsm/kg)	259	282
Urine osmolality	112	286

After discussing the results, the patient admitted to drinking 4-6 liters of water a day to relieve intermittent abdominal pain. When severe, she would drink 6-8 liters of water within an hour. We discussed polydipsia and water intoxication and its detrimental effects on brain and cardiac function. The patient had no more admissions with electrolyte disturbance thereafter.

## Discussion

We have presented the case of a young female who presented to the hospital repeatedly with significant electrolyte derangement. It was only after a thorough history that the underlying cause of this electrolyte disturbance was identified. The patient’s electrolyte disturbance was treated through adequate replacement and then the diagnosis was confirmed through an overnight fluid fast.

Water intoxication leads to a significant electrolyte imbalance. Hypo-osmolality occurs as a result, impairing the kidneys' ability to excrete free water. This contributes to serum dilution and potentially fatal brain disturbance [5].

There are several differential diagnoses to consider when faced with a patient with multiple dyselectrolytemias. The presence of low serum and urine osmolality makes water intoxication a primary differential. A history of eating disorder makes psychogenic polydipsia a secondary differential. Beer potomania due to excessive intake of beer can cause severe hyponatremia but was an unlikely diagnosis, as the patient had no history of alcoholism [6]. Addisonian crisis and diabetic ketoacidosis involve excessive renal electrolyte loss but are accompanied by dehydration and increased urine osmolality; this patient had normal glucose and cortisol levels, ruling out both conditions. Conn’s syndrome, which also causes chronic hypokalemia, was ruled out by normal serum renin and aldosterone levels [7]. Total parenteral nutrition in combination with excess water can cause overhydration and similar electrolyte disturbances. Our patient was not receiving such nutrition [8]. Ecstasy stimulates a severe thirst reaction, as well as the inappropriate secretion of antidiuretic hormone, resulting in excess fluid overload. Our patient had no history nor symptoms of ecstasy ingestion [9]. Diuretic abuse for weight control can cause similar findings but our patient denied such. Finally, syndrome of inappropriate antidiuretic hormone secretion (SIADH) commonly causes hyponatremia but other electrolytes are rarely affected and urine osmolality is usually inappropriately high [3].

Psychogenic polydipsia is a psychiatric condition in which patients feel the need to consume significant volumes of water on a regular basis [2,4]. This rarely causes water intoxication in the presence of normal functioning kidneys. The maximum hourly water excretion rate in normal adults rarely exceeds 0.8-1 liter per hour. Therefore, to become water intoxicated, one has to drink in excess of 0.8-1 liter per hour [5,10]. Signs and symptoms vary depending on the degree of electrolyte disturbance. Mild to moderate hyponatremia may be asymptomatic while moderate to severe hyponatremia can present with nausea, vomiting, seizures, and coma in severe cases [5,10].

The treatment of water intoxication involves fluid restriction to less than 2 liters per day. Diuretics can be used to aid the excretion of excess fluid. The serum sodium levels gradually return to normal. In the case of severe hyponatremia with a neurological disturbance, a more rapid correction with intravenous sodium chloride according to the local protocol will be required. Bringing the serum sodium up by 4-6 mmol/L should be adequate so as to prevent osmotic demyelination syndrome [5,10].

In 1938, Barahal documented the first case of water intoxication in a patient with schizophrenia, and since then, many reports have appeared. In most cases of water intoxication reported in the literature, the fatality is reportedly due to hyponatremia [11-12]. Little is mentioned about accompanying electrolyte disturbances, such as hypomagnesemia, hypokalemia, and hypophosphatemia, which can all cause fatal cardiac arrhythmias. The patient in this case had dilutional dyselectrolytemias involving urea plus six electrolytes, including life-threatening hyponatremia, hypokalemia, hypomagnesemia, and hypophosphatemia.

Our learning points from this case include the concept that water intoxication is common but can easily be a missed cause of dilutional hyponatremia. This diagnosis was missed in a patient with a past medical history of an eating disorder and recurrent admissions. Severe water intoxication can affect multiple electrolytes, which can, in turn, cause life-threatening cardiac arrhythmias. There is a significant list of differential diagnoses for multiple dyselectrolytemias for which a thorough investigation is required. Overall, early detection of water intoxication by taking a detailed history from the patient can identify chronic polydipsia and mitigate the chance of further harm.

## Conclusions

We have presented a case that emphasizes the importance of obtaining an accurate fluid intake history in cases of multiple electrolyte disturbances suspected to be due to water intoxication. Healthcare providers need to be aware that water intoxication is more than just hyponatremia. When faced with similar clinical scenarios and significantly deranged electrolytes, clinicians should consider water intoxication as a potential diagnosis.

## References

[1] Farell DL, Bower L (2003). Fatal water intoxication. J Clin Pathol.

[2] Bhatia MS, Goyal A, Saha R, Doval N (2017). Psychogenic polydipsia - management challenges. Shanghai Arch Psychiatry.

[3] Wakil A, Ng JM, Atkin SL (2011). Investigating hyponatraemia. BMJ.

[4] Krogulska A, Nowicka D, Nowicki Z, Parzecka M, Sakson-Slominska A, Kuczynska R (2019). A loss of consciousness in a teenage girl with anorexia nervosa, due to polydipsia: case report and a minireview. Eat Weight Disord.

[5] Verbalis JR, Goldsmith SR, Greenberg A, Korzelius C, Schrier RW, Sterns RH, Thompson CJ (2013). Diagnosis, evaluation, and treatment of hyponatraemia: expert panel recommendation. Am J Med.

[6] Lodhi MU, Saleem TS, Kuzel AR (2017). "Beer potomania" - a syndrome of severe hyponatremia with unique pathophysiology: case studies and literature review. Cureus.

[7] Stowasser M, Gordon RD, Rutherford JC, Nikwan NZ, Daunt N, Slater GI (2001). Review: diagnosis and management of primary aldosteronsism. J Renin Angiotensin Aldosterone Syst.

[8] Chowdary KV, Reddy PN (2010). Parenteral nutrition: revisited. Indian J Anaesth.

[9] Campbell GA, Rosner MH (2008). The agony of ecstasy: MDMA (3,4-methylenedioxymethamphetamine) and the kidney. Clin J Am Soc Nephrol.

[10] Sahay M, Sahay R (2014). Hyponatremia: a practical approach. Indian J Endocrinol Metab.

[11] Ferrier IN (1985). Water intoxication in patients with psychiatric illness. Br Med J (Clin Res Ed).

[12] Dundas B, Harris M, Narasimhan M (2007). Psychogenic polydipsia review: etiology, differential, and treatment. Curr Psychiatry Rep.

